# Editorial: Biofabrication of nanostructures for environmental, agricultural, and biomedical applications

**DOI:** 10.3389/fchem.2023.1283676

**Published:** 2023-09-20

**Authors:** Sougata Ghosh, Raymond J. Turner, Sirikanjana Thongmee

**Affiliations:** ^1^ Department of Microbiology, School of Science, RK University, Rajkot, India; ^2^ Department of Physics, Faculty of Science, Kasetsart University, Bangkok, Thailand; ^3^ Department of Biological Sciences, University of Calgary, Calgary, Canada

**Keywords:** biogenic nanoparticles, metabolites, functionalization, photocatalysis, biocontrol

The Research Topic entitled “Biofabrication of Nanostructures for Environmental, Agricultural, and Biomedical Applications” was dedicated to original research and reviews articles on bio-inspired processes for fabrication of nanomaterials for antimicrobial, antibiofilm, anticancer, tissue engineering, dye degrading and biocontrol applications. Various bacteria, fungi, algae, medicinal plants their metabolites such as lignin, silbinin, curcumin, play active role in the synthesis of the nanoparticles and their stabilization. Challenges exist using biological materials to synthesize nanostructures compared to physical-chemical techniques. This comes from the complexity of the resulting biochemical mixture and limitations within the physical chemical parameters of the synthesis. However, the challenges become advantages as the biomolecules involved in the biogenic synthesis often bestow unique and superior properties. The biogenic nanoparticles tend to be more biocompatible and are thus considered as ideal candidate to design nanomedicine (Patil and Chandrasekaran, 2020). Here in this issue, we see creative examples in using biomaterials to create unique natural materials, and nano structures towards interesting applications.

Bilal et al. developed novel guided tissue regeneration (GTR) membranes from mucilage extracted from the chia seed, Lignin@ZnO, and polyvinyl alcohol (PVA). The ZnONPs were irregular with size lesser than 50 nm. The membrane exhibited randomly interconnected structures, with smooth morphology with Lignin@ZnO. The GTR showed satisfactory swelling and mechanical properties while the degradation started after 24 h. Further, superior antibacterial activity of the GTR was reported against *Staphylococcus aureus* and *Escherichia coli*.

In another study, Ravi et al. developed silibinin conjugated gold nanoparticles (Sb-GNPs) for anticancer applications against lung carcinoma cell line (A549). The hydrodynamic size of GNPs and Sb-GNPs were 107 ± 9 nm and 163 ± 5 nm, respectively while the zeta potentials were −19.6 mV ± 0.648 and −22.2 mV ± 0.458 mV, respectively. The pH responsive release of silibinin was confirmed to be facilitated by an acidic pH up to 200 min. The IC50 value of Sb-GNPs was 4.8 μM (w.r.t. Sb concentration) while it was 24.8 μM for the free silibinin.

Bloch et al. reported phytofabrication of zinc oxide particles (ZnOPs) and silver mixed zinc oxide particles (ZnOAg1Ps, ZnOAg10Ps, ZnO10Ag1Ps) using *Plumbago auriculata* leaf extract (PALE) by varying the concentration of the metal precursor salts, i.e., zinc acetate and silver nitrate. It was speculated that the phytochemicals such as polyphenols, flavonoids, reducing sugar, starch, citric acid and plumbagin might have played a significant role in the synthesis and stabilization of the nanoparticles that varied in size from 90 to 400 nm, approximately. The nanocomposite (ZnOAg10Ps) exhibited 95.7% photocatalytic degradation of methylene blue with a rate constant of 0.0463 s^−1^ that followed a first order kinetics.

Tawre et al. used *Curcuma aromatic* rhizome extract for synthesis of silver nanoparticles (CAAgNPs) with efficient antibacterial, antibiofilm and synergistic effects against multidrug-resistant (MDR) pathogens. The nanoparticles were spherical and monodispersed with size around 13 ± 5 nm. The minimum inhibitory concentrations (MICs), minimum bactericidal concentrations (MBCs) and minimum biofilm inhibitory concentrations (MBICs) of CAAgNPs against *Pseudomonas aeruginosa*, NCIM 5029 and PAW1, and, *S. aureus*, NCIM 5021 and S8 were in range from 8 to 128 μg/mL. Pronounced biofilm disruption (50%) and antimicrobial synergy with antibiotics was noted against *P. aeruginosa* PAW1 and *S. aureus* S8.

Yet in another study by Ghosh et al. *T. cordifolia* leaf extract was used for the synthesis of AgNPs with the size ranging from 43.82 ± 1.023 nm to 91.28 ± 1.12 nm. Both *Tinospora cordifolia* extract and the AgNPs inhibited the bacteria with the zone of inhibition equivalent to 10–15 mm and 12–18 mm, respectively. The AgNPs inhibited the bacterial biofilm which was speculated to be the result of directly interaction as well as reduction of biofilm associated polysaccharides, lipids, and nucleic acids.

The mini-review by Ngcongco et al. discusses the advances in bacteria, fungi, and plant mediated synthesis metallic nanoparticles with enzymatic activity identical to that of peroxidase, haloperoxidase, oxidase, catalase, hydrolase, and superoxide dismutase. Further, the vital aspects such as toxicity, mechanism and patents in the area of the nanozymes are also covered.

An interesting research by Malhotra et al. reports synthesis of ZnONPs employing an ethanolic extract of *Eupatorium odoratum*. The phytogenic nanoparticles revealed a hexagonal phase with wurtzite structure with a particle size of ∼50 nm that significantly inhibited bacterial pathogens with high killing efficacy (99.99%) at 500 μg/mL concentration. Further, the commercial central venous catheters (CVCs) coated with the phytogenic ZnONPs could significantly resist the biofilm formation by *P. aeruginosa*, *E. coli* and *S. aureus*.

Singh et al. employed *Serratia* sp. ZTB29 strain for bacteriogenic fabrication of spherical copper oxide nanoparticles (CuO-NPs) with an average size of 22 nm. It was speculated that the bacterial metabolites with the ester (C=O), carboxyl (C=O), amine (NH), thiol (S-H), hydroxyl (OH), alkyne (C-H), and aromatic amine (C-N) groups played a key role in the synthesis and stabilization of the CuO-NPs. The nanoparticles could significantly inhibit phytopathogenic bacteria and fungus, such as *Xanthomonas* sp. and *Alternaria* sp., respectively. It is important to note that the treatment with CuO-NPs also improved the growth characteristics of maize plants that support its agricultural applications.

Trzcińska-Wencel et al. reported mycogenic AgNPs and ZnONPs using *Fusarium solani* IOR 825. The AgNPs were spherical with a size of 8.27 nm while the ZnONPs were larger in size (117.79 and 175.12 nm). The nanoparticles inhibited bacterial pathogens, namely, *Agrobacterium tumefaciens* IOR 911, *Pectobacterium carotovorum* PCM 2056, *Pseudomonas syringae* IOR 2188, *Xanthomonas campestris* IOR 512. Also, both AgNPs and ZnONPs inhibited the mycelial growth of *Alternaria alternata, Fusarium culmorum, Fusarium oxysporum, Phoma lingam,* and *Sclerotinia sclerotiorum*. Interestingly, the mycogenic AgNPs exhibited a sterilization effect on maize seeds while ZnONPs promoted the growth of the seedlings with notable improvement in the fresh and dry biomass.

The content of this Research Topic can be summarized as use of biogenic nanomaterials with attractive physicochemical and optoelectronic properties for inhibition of bacteria, fungi, cancer along with environmental and agricultural applications.

Control over the geometry of the nanoparticles can result in the desired optoelectronic and physicochemical properties. Although biogenic nanoparticles are non-toxic and environmentally safe, optimized synthesis methods must be designed. Despite being sustainable and environmentally friendly, biogenic fabrication is often time-consuming because of the cultivation time required to get adequate microbial biomass. Moreover, they are not monodispersed. These challenges can be addressed by rational selection of microorganisms, optimization synthesis parameters such as duration, pH, temperature, concentration of precursors, culture age, and cell density. This may allow scale up for large-scale production of nanoparticles.

Another important aspect is the recovery of the intracellularly synthesized nanoparticles. While it is easier to recover the extracellular nanoparticles by employing either filtration or centrifugation, recovery of the intracellularly produced nanoparticles may involve cell disruption by physical, chemical, and/or biological methods. The cells can be disrupted by ultrasonication, freeze-thaw, SDS, NaOH treatment for release of the nanoparticles from the cell interior. Detailed mechanism for the synthesis can be deciphered by integrated approach using genomics, proteomics and metabolomics. Further, genetically modified microbes can be developed with high nanobiotechnological potential with desired control over shape and size. The functionalization studies will help to understand the surface modification, drug loading efficiency, drug release kinetics, targeted delivery, drug uptake, accumulation, and stability that will help to develop novel nanomedicine. In view of the background, this Research Topic will enable the rational development of bioprocesses for synthesis, recovery, modification and application of tailor made nanostructures using bacteria, fungi, plants and their metabolites.
